# Spatial pattern and temporal trend of mortality due to tuberculosis^10^

**DOI:** 10.1590/1518-8345.2049.2992

**Published:** 2018-05-07

**Authors:** Ana Angélica Rêgo de Queiroz, Thaís Zamboni Berra, Maria Concebida da Cunha Garcia, Marcela Paschoal Popolin, Aylana de Souza Belchior, Mellina Yamamura, Danielle Talita dos Santos, Luiz Henrique Arroyo, Ricardo Alexandre Arcêncio

**Affiliations:** 1 PhD. Escola de Enfermagem de Ribeirão Preto, Universidade de São Paulo, PAHO/WHO Collaborating Centre for Nursing, Ribeirão Preto, SP, Brazil. Coordenação de Aperfeiçoamento de Pessoal de Nível Superior (CAPES).; 2 Master’s student. Escola de Enfermagem de Ribeirão Preto, Universidade de São Paulo, PAHO/WHO Collaborating Centre for Nursing, Ribeirão Preto, SP, Brazil. Coordenação de Aperfeiçoamento de Pessoal de Nível Superior (CAPES).; 3 PhD. Escola de Enfermagem de Ribeirão Preto, Universidade de São Paulo, PAHO/WHO Collaborating Centre for Nursing, Ribeirão Preto, SP, Brazil. Fundação de Amparo à Pesquisa do Estado de São Paulo (FAPESP).; 4 PhD. Escola de Enfermagem de Ribeirão Preto, Universidade de São Paulo, PAHO/WHO Collaborating Centre for Nursing, Ribeirão Preto, SP, Brazil. Fundação de Amparo à Pesquisa do Estado de São Paulo (FAPESP).; 5 Doctoral student. Escola de Enfermagem de Ribeirão Preto, Universidade de São Paulo, PAHO/WHO Collaborating Centre for Nursing, Ribeirão Preto, SP, Brazil. Fundação de Amparo à Pesquisa do Estado do Amazonas (FAPEAM).; 6 Post-doctoral fellow. Escola de Enfermagem de Ribeirão Preto, Universidade de São Paulo, PAHO/WHO Collaborating Centre for Nursing, Ribeirão Preto, SP, Brazil. Programa Nacional de Pós Doutorado (PNPD) - Coordenação de Aperfeiçoamento de Pessoal de Nível Superior (CAPES).; 7 Doctoral student. Escola de Enfermagem de Ribeirão Preto, Universidade de São Paulo, PAHO/WHO Collaborating Centre for Nursing, Ribeirão Preto, SP, Brazil. Coordenação de Aperfeiçoamento de Pessoal de Nível Superior (CAPES).; 8 Doctoral student. Escola de Enfermagem de Ribeirão Preto, Universidade de São Paulo, Ribeirão Preto, SP, Brazil. Fundação de Amparo à Pesquisa do Estado de São Paulo (FAPESP).; 9 PhD. Associate Professor, Escola de Enfermagem de Ribeirão Preto, Universidade de São Paulo, PAHO/WHO Collaborating Centre for Nursing, Ribeirão Preto, SP, Brazil.

**Keywords:** Tuberculosis, Mortality, Spatial Analysis, Time Series Studies, Health Information Systems, Geographic Information Systems

## Abstract

**Objectives:**

To describe the epidemiological profile of mortality due to tuberculosis (TB), to analyze the spatial pattern of these deaths and to investigate the temporal trend in mortality due to tuberculosis in Northeast Brazil.

**Methods:**

An ecological study based on secondary mortality data. Deaths due to TB were included in the study. Descriptive statistics were calculated and gross mortality rates were estimated and smoothed by the Local Empirical Bayesian Method. Prais-Winsten’s regression was used to analyze the temporal trend in the TB mortality coefficients. The Kernel density technique was used to analyze the spatial distribution of TB mortality.

**Results:**

Tuberculosis was implicated in 236 deaths. The burden of tuberculosis deaths was higher amongst males, single people and people of mixed ethnicity, and the mean age at death was 51 years. TB deaths were clustered in the East, West and North health districts, and the tuberculosis mortality coefficient remained stable throughout the study period.

**Conclusions:**

Analyses of the spatial pattern and temporal trend in mortality revealed that certain areas have higher TB mortality rates, and should therefore be prioritized in public health interventions targeting the disease.

## Introduction

Tuberculosis (TB) is a global public health problem and the main cause of death due to infectious disease^1^. In Brazil, the incidence of TB was around 41.0 cases per 100,000 inhabitants in 2015, and the mortality rate was 2.4 deaths per 100,000 inhabitants.

In the last two decades there has been a decline in the incidence of TB and the TB mortality rate, although the disease still influences the economy and the health systems. In 2014, in accordance with the Sustainable Development Goals, the World Health Organization (WHO) established the ‘End TB strategy’, whose target is to reduce TB mortality by 95.0% and TB incidence by 90.0% relative to the 2015 figures by 2035^1^ ^-^ ^2^. 

There are barriers to achieving these targets; namely the need for new, sustainable diagnostic technologies, new drugs that shorten the treatment time and healthcare systems structured in such a way that it can reach all populations affected by the disease. 

Several studies have assessed the factors associated with TB mortality, identifying factors related to the patient’s clinical condition such as multiple drug resistance (TB-MDR), infection with the human immunodeficiency virus (HIV), malnutrition, diabetes and its association with hepatitis. Gender is also factor, as mortality is higher in men^3^. In addition, cultural factors come into play, for example the social stigma attached to the disease, which means that individuals avoid attending clinics for fear of receiving a TB diagnosis, and then search for alternative therapies^4^. The efficiency and quality of healthcare delivery also influence TB mortality^5^ ^-^ ^6^. 

Due to the available diagnostic and treatment technologies, TB deaths can be considered an unfair event, which highlights the need to reduce its occurrence. Thus, in order to reduce the mortality rate due to TB in Brazil, social as well as political, human and economic changes are needed. 

Spatial and temporal patterns in TB mortality have been studied^7^ ^-^ ^8^, but have attracted less research attention than TB incidence or prevalence. An important gap in the literature on TB mortality trends is that there is no published evidence on whether Brazil will meet the 2015 goal for the reduction in TB deaths, mainly in Northeast Brazil. 

TB mortality is an important issue in nursing, because TB nurses have to select appropriate methods for controlling TB and ensuring that TB patients complete therapy successfully. If vulnerable areas to TB deaths are evidenced in reports, TB nurses can act as an important professional in the development of actions to control TB in these places.

Thus, the study aimed to describe the epidemiological profile of mortality due to tuberculosis (TB), to analyze the spatial pattern of these deaths and to investigate the temporal trend in mortality due to tuberculosis in Northeast Brazil

## Methods

**Figure 1:**
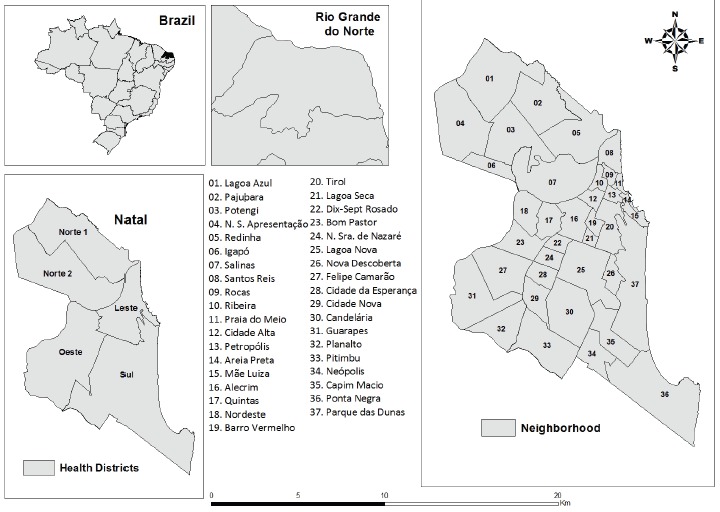
Geographical location of the municipality under study and its division according to health districts and neighborhoods, Natal, RN, Brazil.

This was an ecological study^9^. The study was carried out in Natal, the capital of the state of Rio Grande do Norte, in Northeast Brazil. The city is divided into 36 neighborhoods and one area of environmental reserve and healthcare is administered through five health districts: North I, North II, South, East and West^10^ ^)^ (Figure 1). The city has a Human Development Index (HDI) of 0.7 and a Social Exclusion Index (SEI) of about 0.6, a Poverty Index of 40.86% and Gini Index of 0.6^11^.

The justification for choosing Natal as the study context is that the Ministry of Health has made it a priority city in relation to TB control. In 2015, the incidence of TB in Natal was 37.1 cases per 100,000 inhabitants and the mortality rate was 2.6 deaths per 100,000 inhabitants according to local data from Municipal Health Secretary of the city. 

The study population consisted of all cases of death due to TB for which an underlying and associated cause were registered in the Mortality Information System (MIS) between 2008 and 2014.

The study data were collected from death certificates (DC), where the underlying or associated cause of death was any clinical form of TB described according to the International Classification of Diseases version 10 (ICD10) codes A15.0 A19.0, and the death was registered in the MIS of the Natal Municipal Health Department-RN. The Epidemiological Surveillance Division of the Natal Municipal Health Department-RN provided the data. After preliminary contact with the city’s MIS coordinator, a date and time at which the data would be collected was scheduled. The data were collected in March 2015.

The socio-demographic variables studied were: age, gender, ethnic background, marital status, education, and occupation. The operational variables were: place of death and medical care.

Descriptive statistics were used to describe the profile of the patients for whom TB was listed as the underlying or associated cause of death. Relative or absolute frequencies were calculated for the categorical variables and position and dispersion measures for the continuous variables. 

The cases were geocoded using TerraView version 4.2.2 software to enable analysis of the spatial distribution of deaths. Next, the addresses of cases in urban Natal-RN were standardized and matched using a digital address segment map in Latlong/WGS84 projection. The units of analysis were city neighborhood and census sector.

First we carried out a point density analysis using the Kernel estimation method, an exploratory interpolation method based on defining circular areas of influence around the points where a phenomenon occurs, and then used these to produce a density map that identifies vulnerable areas^12^ ^-^ ^13^. The density map provides an overview of the disease distribution and can be used to guide exploration of the point pattern of the health data. Therefore, a radius of 1,000 meters was considered. The density distribution maps of TB deaths were produced using ArcGIS 10.2 software.

Next, the mortality rates due to TB were standardized in each neighborhood per gender and age range using the direct method^9^ and considering Natal’s population as the standard. The age ranges chosen were based on the disease distribution in the study population: zero to 15 years, 16 to 59 years and 60 years or older.

Annual TB mortality rates were smoothed using a local empirical Bayes model, with a view to minimize the instability caused by oscillations in small population and underreporting of TB deaths. As a result of applying this method, a weighted average was obtained between the gross rate of the neighborhoods and taking the regional rate of the closest neighbors for reference. This rate considered the population density and the local mean rate, starting from a spatial proximity matrix^14^. Terraview version 4.2.2 was used to calculate the smoothed rates. Next, ArcGIS version 10.2 was used to produce distribution maps of the local empirical Bayes rates, grouped by quintile.

In addition, coefficients for mortality due to TB were expressed as logarithms in order to classify the temporal trend in the disease between 2008 and 2014 as downward, stationary or upward. The time series statistics in StataSE 13 were used for calculating this, applying the Prais-Winsten generalized linear regression method. This procedure corrects the first-order temporal autocorrelation in analyses of organized time series. The annual variance in the measure and its 95% confidence intervals (95%CI) were also calculated^15^.

Approval for the study was obtained from the Research Ethics Committee at the University of São Paulo at Ribeirão Preto College of Nursing, under CAAE (Certificate of Presentation for Ethical Appreciation) 41398915.6.0000.5393.

## Results

During the study period there were 236 deaths in the study area, and TB was registered as the underlying cause in 154 (65.25%) of them. The minimum age at death was eight years and the maximum was 101 years; age was stratified by group (0 14 years; 15 59 years; ≥ 60 years).

The predominant clinical form of TB was pulmonary tuberculosis, without bacteriological or histological confirmation (ICD-10 16.2), specified in 130 cases where TB was the underlying cause of death (84.41%) and 66 cases where it was an associated cause of death (80.49%). In cases where TB was an associated cause of death, the most common underlying cause was infectious and parasitic diseases (n *=* 51, 62.19%), and most often HIV.

Table 1 presents a comparison of the sociodemographic profiles of people for whom TB was listed as the underlying cause of death and those for whom another underlying cause of death was listed.

**Table 1:** Distribution of sociodemographic and clinical characteristics according to the cause of TB mortality. Natal, RN, Brazil, 2015.

Characteristics	TB* mortality			
	Underlying cause		Associated cause	
	n	(%)	n	(%)
Age in years (n=236)				
014	0	0.00	1	1.20
1559	94	61.03	61	74.40
≥ 60	60	38.97	20	24.40
Gender (n=236)				
Male	115	74.70	56	68.30
Female	39	25.30	26	31.70
Ethnicity (n=208)				
Mixed	78	58.20	47	63.50
White	45	33.60	23	31.10
Black	11	8.20	4	5.40
Marital status (n=221)				
Single	81	55.90	44	57.90
Married	47	32.40	23	30.30
Widowed	13	9.00	5	6.60
Divorced	3	2.00	3	3.90
Fixed partner	1	0.70	1	1.30
Education (n=159)				
No education	8	7.60	0	0.00
Unfinished primary education (1 - 3 years)	28	26.70	4	7.40
Finished primary education (4 - 7 years)	32	30.50	17	31.50
Secondary education (8 - 11 years)	22	20.90	17	31.50
Unfinished higher Education (12 - 14 years)	10	9.50	13	24.00
Finished higher education (Over 15 years)	5	4.80	3	5.60
Place of death (n=235)				
Hospital	127	83.00	76	92.70
Other health services	2	1.30	0	0.00
Home	20	13.05	5	6.10
Public road	1	0.65	1	1.20
Others	3	2.00	0	0.00
Medical care (n=163)				
Yes	90	87.40	56	93.30
No	13	12.60	4	6.70

* TB - Tuberculosis

Taking the sample as a whole, 223 (94.50%) deaths were geocoded, and 215 of which were identified in the cartographic database and processed in TerraView version 4.2.2; the remaining 8 cases were processed using the Batch Geocode tool. Losses in the geocoding process were due to inconsistent addresses in the MIS.

**Figure 2:**
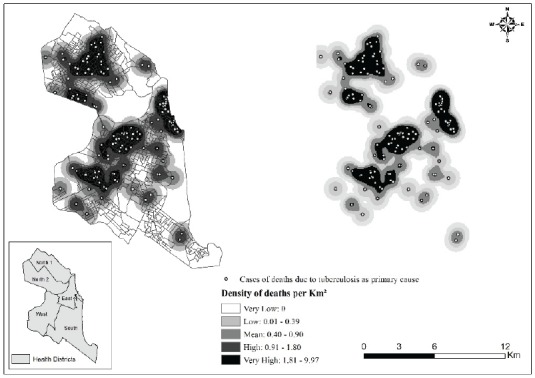
Density distribution of deaths for which tuberculosis was listed as the underlying cause, Natal, RN, Brazil, 2015

**Figure 3:**
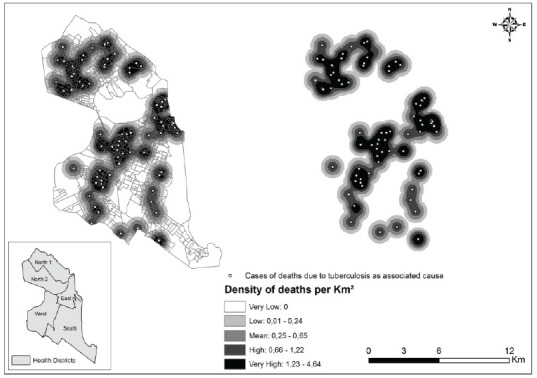
Density distribution of deaths for which tuberculosis was listed as an associated cause, Natal, RN, Brazil, 2015

Figures 2 and 3 respectively represent density maps for deaths in Natal, RN, for which TB was listed as an underlying or associated cause. 

Regarding the standardized rates of death per neighborhood, the annual average mortality rate of TB mortality in Natal during the seven-year study period was 2.74 cases per 100,000 inhabitants, with a higher rate in the Areia Preta neighborhood (16.71 cases/100,000 inhabitants). After correction by the local empirical Bayes method, the neighborhood with the highest rate was Praia do Meio (8.53 cases/100,000 inhabitants). Both Areia Preta and Praia do Meio are in the Eastern health district of Natal. Based on the standardized rates for mortality with TB as an associated cause, the neighborhood with the highest coefficient was Areia Preta (7.30 cases/100,000 inhabitants). After correction by the local empirical Bayes method, the highest rates were found in the Mãe Luiza neighborhood (4.19 cases/100,000 inhabitants); once again, both these neighborhoods were located in the Eastern health district.

**Figure 4:**
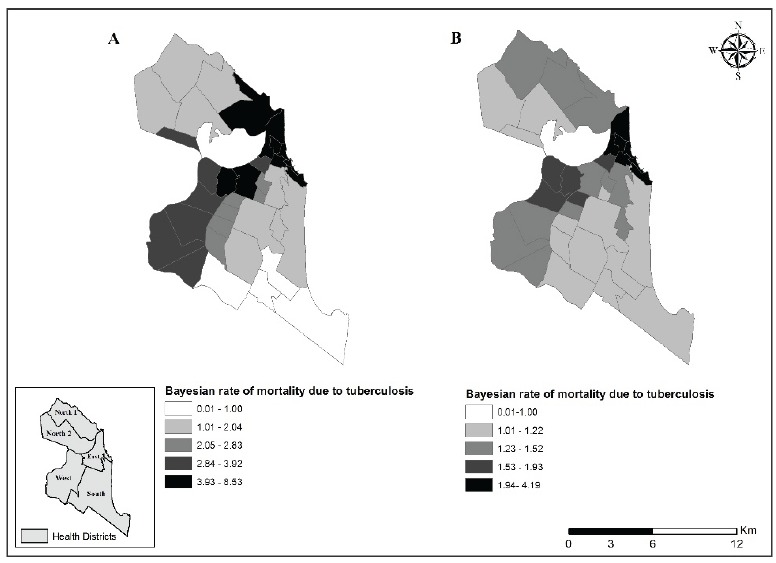
Distribution of annual local empirical Bayesian rates of mortality with tuberculosis as the underlying cause (A) or an associated cause (B). Figures are deaths per 100,000 inhabitants per year for neighborhoods of Natal, RN, Brazil, 2015.

Figure 4 displays the spatial distribution of the annual local empirical Bayesian rates of mortality with TB as an underlying or associated cause. 

During the study period, the gross TB mortality rate (underlying and associated cause) in the city of Natal was 5.25 per 100,000 inhabitants in 2008, and 4.00 per 100,000 inhabitants in 2014. The coefficient for mortality due to TB (underlying or associated cause) remained stable during the study period, with annual variation of -2.2% (CI95% -4.8%; -0.3%). 

## Discussion

The study aimed to describe the epidemiological profile of TB mortality, to analyze the spatial pattern of these deaths and to investigate the temporal trend in TB mortality in Northeast Brazil.

The study showed that in most of the cases where TB was listed as the cause of death, the deceased was male, between 15 and 59 years of age, single and of mixed ethnicity. In spatial terms, deaths from TB were concentrated in the West, North and East health districts. The TB mortality rate remained stable throughout the study period, with annual variation of -2.2% (CI95% -4.8%; -0.3%).

With regard to the epidemiological profile, the results do not differ from those of other studies of TB mortality in Brazil and around the world^6^. The gender distribution of mortality (a higher proportion of deaths in males) is similar to the incidence pattern of the disease, with higher morbidity among men^6^ and people of mixed ethnicity^3^. This may be due to the relationship between biological and social factors, or to gender differences in exposure factors and prevalence of infection with evolution to disease, amongst other issues related to access to health services.

The study showed that most of the cases had a low level of education. Other authors have reported that low education, unemployment and income are individual-level factors associated with increased incidence of TB and with lower treatment compliance, and therefore they may also be related to access to health services and the quality of diagnosis. People with less education and lower incomes are less likely to perceive that they are at risk and to comply with the treatment, because they present individual and unequal access to information, to benefits deriving from knowledge, to consumer goods and mainly to health services^16^ ^).^

Moreover, regarding the age of TB mortality as a basic and associated cause, the disease affected patients who were in the economically active age range (15 to 59 years), a finding which is in line with another Brazilian study^17^. This is an important issue, since TB mortality affects economic and social development at a regional level, and is both a cause and consequence of poverty^18^. Regarding an associate cause of TB mortality, AIDS cases in Brazil are higher in individuals between 25 and 39 years of age for men and women^16^. Furthermore, an earlier study showed a larger number of cases of TB/HIV co-infection among individuals between 30 and 50 years of age.

As far as the operational profile is concerned, the largest proportion of deaths happened in hospitals to patients who had been receiving medical care before death. This indicates the weakness of Primary Health Care (PHC) in terms of problem-solving and ability to respond to the needs of TB patients, as this healthcare level should serve as the point of entry to healthcare services, giving patients the opportunity to receive an early diagnosis, and hence a better prognosis. 

The most frequent clinical form of TB in our sample was pulmonary TB, without bacteriological or histological confirmation; this is in line with WHO estimates reporting that the mean occurrence of pulmonary TB is about 85%^1^. The general lack of bacteriological or histological confirmation raises concerns about the reliability of TB diagnoses; bacteriological tests, such as sputum smears and cultures available in the public network of the Brazilian Unified Health System (UHS) possibly in association with bronchoscopy or biopsy, should be used to confirm TB diagnoses^6^.

Another aspect verified in the study was the mortality due to the association between TB and HIV. TB is the most frequent opportunistic disease in HIV patients, and several studies have demonstrated that it is also one of the main causes associated with death in that population. A study carried out in Africa^19^ reported that 47.8% of all deaths investigated were related to TB/HIV co-infection, which is consistent with global statistics showing that TB is the cause of death in one out of three Acquired Immune Deficiency Syndrome (AIDS) patients.

It has been shown that characteristics related to treatment history such as treatment abandonment, multiple drug resistance and TB/HIV co-infection are associated with the death of TB cases^(^^20)^.

In contrast, death from TB may be considered an unfair and avoidable event, as the UHS has all the resources required to diagnose and treat patients, and treatment is freely and universally available^21^. An important issue is whether all Brazilian populations affected by TB have access to care; some groups, mostly vulnerable groups (homeless people, prisoners, drugs users and the unemployed, amongst others) still face many barriers to care? ^22^. 

Considering the Universal health system has been adopted in Brazil under a social right perspective and equality, it would be mandatory to provide actions or TB care according to the needs of the population. Health equality is an important index to verify when each individual has a fair opportunity to achieve his or her full health potential^23^. When differential mortality can be linked to differences in social conditions, it is clear that health equality has not been achieved^21^. TB mortality in Brazil is more seriously affected by social inequality than by the availability of medical technology for diagnosis and treatment^1^. 

The spatial distribution of TB is affected by socioeconomic inequalities across the study area. In this study, it could be proven that the space was relevant in investigating and understanding the occurrence and distribution of mortality in the city, as it is the environment where the infectious agent circulates, and which, under specific conditions, provokes the disease and even death as a result^24^.

The spatial distribution of cases indicated that TB mortality (TB as the underlying or associated cause of death) was most frequent in certain neighborhoods of the East and West health districts. There were fewer TB-related deaths in the South region. Inspection of the density distribution maps of the produced points revealed that TB-related deaths were unevenly distributed across the city, with ‘hot spots’ in the East, West and Northern health districts. 

The more intense point clusters in the North shown in Figures 2 and 3 can be explained by the fact that the kernel density analysis is based on the counting of points per km^2^ within influential circulation areas, weighted by the distance of each from the location of interest, without considering the population of the areas^12^ ^-^ ^13^.

The neighborhoods in the West and North health districts where TB mortality was highest coincided with the regions of the city with the worst social indicators and are areas where incomes are generally low, typically areas on the outskirts of the city. 

The municipality has organized its health care services by heath district, and for each health district there are enough numbers of services to meet the main needs of the population. The North health district is highly populated, representing 37.77% of the population of municipality and concentrating 40% of the slums and population living on a monthly income below the minimum wage^25^; it has coverage by the Family Health Strategy (FHS) of about 77.00% of the population in North I, and North II has 63.00% of the population covered by the FHS^10^. 

The West health district is classified as the poorest based on family income data, and it is also the second most populous and has the highest density of TB cases^25^. In addition, it has the highest number of people per household, concentrating the largest number of subnormal clusters, the second highest percentage of slums^25^ and 69.00% of the population have FHS coverage^10^.

The East health district is part of a region with better social indicators, although some neighborhoods (Rocas, Praia do Meio, Santos Reis and Mãe Luiza) have social indices comparable to those typical of the North and West, and have the highest incidence and TB mortality of anywhere in the city^10^. In addition, a feature of the East regions is the social inequality, as areas of low social vulnerability sit very close to areas of high social vulnerability^25^. About 37.00% of the population has FHS coverage in the East health district, which may represent weakness in terms of TB care either for diagnosis or treatment^10^.

These data show that TB mortality mainly affects the health districts with the worst HDI values, indicating that socioeconomic factors play an important role on the impact of TB.

There is a negative association between HDI and the impact of TB, and the spatial distribution of the disease is affected by multiple factors including the territorial extent, disordered population growth and the concentration of people on the outskirts. In this sense, the geographical space reveals the symbolic dimension of the social relationships, in which the factors associated with the development and dissemination of diseases are expressed, as well as its distribution among the different social groups^20^.

There is a global downward trend in TB mortality. The number of cases in Brazil has dropped by 2% per year on average over the last ten years^24^, which suggests that mortality rates in Brazil follow the WHO proposal concerning the priorities in terms of early case detection, patient treatment and its conclusion with cure as the outcome^2^. 

According to the WHO’s Global Tuberculosis Report^1^, Brazil reached all the TB-related millennium development goals (stop and reverse the upward trend in TB incidence coefficient by 2015), as well as the ‘Stop TB Partnership’ target of reducing the prevalence of TB and TB mortality by 50% relative to the 1990 figures by 2015.

However, in order to achieve the WHO goal of reducing TB deaths by 75% by 2025 and 95% by 2035 through the ‘End TB strategy’, the incidence rate would have to drop by 10% per year for the next 20 years^24^.

Various strategies have been proposed to improve Brazilian TB patients’ access to healthcare, and it has been suggested that healthcare decentralization is the best way of achieving health equality and equal access to care for all social strata. Thus, the literature has shown FHS advances in relation to qualification of the system, the regional specificity of FHS policies and programs that represent a way of tackling the fragmented nature of the healthcare system, but as yet it has not had an impact on disease rates^27^.

The limitations of this study include the use of secondary data derived from the MIS, as there are weaknesses in these data; for example, underreporting of TB and non-completion of some fields in the DC, which may bias the results of the analyses. Further studies are needed to determine the statistical relationship between TB mortality and social variables. The issue regarding the data collection period could be a potential limitation of the study (March 2015), since the total number of TB deaths occurred by 2014 may still not have been up-to-date, which could influence the findings, mainly in the analysis of temporal series. 

## Conclusion

This study contributes to knowledge on TB mortality by describing the progress Brazil has made towards achieving the ‘End TB’ targets. Thus, the results of the study through the Geographic Information System technologies evidence relevant aspects to Nursing in terms of care planning and implementation, mainly in the areas with higher death rates. These results may contribute to improve the quality of care by nurses in the Primary Health Care. In addition, the results of the study may not only be relevant to local management, but also to other contexts with a similar epidemiological profile with respect to TB. 
